# The Role of Monoaminergic Neurotransmission for Metabolic Control in the Fruit Fly *Drosophila Melanogaster*

**DOI:** 10.3389/fnsys.2017.00060

**Published:** 2017-08-22

**Authors:** Yong Li, Lasse Tiedemann, Jakob von Frieling, Stella Nolte, Samar El-Kholy, Flora Stephano, Christoph Gelhaus, Iris Bruchhaus, Christine Fink, Thomas Roeder

**Affiliations:** ^1^Laboratory of Molecular Physiology, Department of Zoology, Kiel University Kiel, Germany; ^2^Department of Molecular Parasitology, Bernhard-Nocht-Institute for Tropical Medicine Hamburg, Germany; ^3^German Center for Lung Research (DZL), Airway Research Center North (ARCN) Kiel, Germany

**Keywords:** octopamine receptor, tyramine receptor, insulin, body fat distribution, insulin release

## Abstract

Hormones control various metabolic traits comprising fat deposition or starvation resistance. Here we show that two invertebrate neurohormones, octopamine (OA) and tyramine (TA) as well as their associated receptors, had a major impact on these metabolic traits. Animals devoid of the monoamine OA develop a severe obesity phenotype. Using flies defective in the expression of receptors for OA and TA, we aimed to decipher the contributions of single receptors for these metabolic phenotypes. Whereas those animals impaired in *octß1r*, *octß*2r and *tar1* share the obesity phenotype of OA-deficient (*tβh*-deficient) animals, the *octß*1r, *octß*2r deficient flies showed reduced insulin release, which is opposed to the situation found in *tβh*-deficient animals. On the other hand, OAMB deficient flies were leaner than controls, implying that the regulation of this phenotype is more complex than anticipated. Other phenotypes seen in *tβh-deficient* animals, such as the reduced ability to perform complex movements tasks can mainly be attributed to the *octß*2r. Tissue-specific RNAi experiments revealed a very complex interorgan communication leading to the different metabolic phenotypes observed in OA or OA and TA-deficient flies.

## Introduction

Hormones are known to have a major impact on various metabolic traits. Among these hormones biogenic amines take a special position as they modulate these metabolic traits at different levels. Two of these amines, octopamine (OA) and tyramine (TA) are specifically relevant in invertebrates (Roeder, 1999, 2005). They act as functional equivalents of the vertebrate hormones/transmitters epinephrine and norepinephrine; similar to their roles in vertebrates, in which epinephrine- or norepinephrine-mediated signaling leads to a variety of metabolic changes (Debuyser et al., 1991; Bachman et al., 2002), OA and TA appear to be similarly potent in order to control metabolic traits in invertebrates (Lange, 2009; Li et al., 2016). Although TA and OA have been shown to act as independent neuroactive compounds, they share a large number of similarities (Roeder et al., 2003; Saraswati et al., 2004; Lange, 2009). Most importantly, OA producing cells always contain TA, as the latter one serves as a biological precursor for OA (Roeder, 2002, 2005; Cole et al., 2005). On the other hand, only very few neurons in the insect brain produce TA but no OA, making it hard to examine how the different actions of both compounds are disentangled under physiological conditions (Monastirioti et al., 1995; Busch et al., 2009; Selcho et al., 2014). Beside the countless modulatory actions in invertebrates that can be attributed to these monoamines (Roeder, 1999, 2002), they have also been shown to regulate various metabolic traits. OA in particular appears to take a central position in regulating metabolism associated traits. It was shown that OA signaling is highly relevant in controlling behaviors with a direct impact on energy expenditure comprising the regulation of physical activity or the timing of sleep. Moreover, it was shown recently that OA directly affects the metabolic resting rate, therewith directly influencing fat storage (Li et al., 2016).

Although this role of both monoamines has mainly been studied in fruit flies, it appears also to apply to other insect and even to other invertebrates such as nematodes (Suo et al., 2006). As already mentioned, the vertebrate counterparts epinephrine and norepinephrine act in very similar ways as OA and TA do. In both systems, the corresponding hormones are released in times of stress and act as major transducers that orchestrate the organism’s stress reaction (Atgié et al., 1998; Adamo and Baker, 2011; Even et al., 2012). Release of these compounds should thus increase physical activity and resting metabolic rates reducing body fat stores. Diminished release of these compounds has exactly the opposite effects; it reduces activity and the metabolic rate, which leads, long-term, to more body fat.

Release of OA and TA modulates various behaviors and metabolic traits in a well-coordinated manner in order to shift the animal’s physiology to a high performance, high energy-expenditure state (Li et al., 2016). Thus, they appear to take a central position in the regulatory network responsible for inter-organ communication (Rajan and Perrimon, 2011). This comprises both types of behaviors, those that are associated with energy intake as well as those associated with energy expenditure. Food intake as the only energy source is also under the control of OA-mediated signaling (Zhang et al., 2013). Directly associated with this effects is the enhanced physical activity that is seen during periods of starvation, which appears to be devoted to enable efficient searches for novel food sources (Yang et al., 2015). OA and TA control movement activity and movement performance at different levels. In larval muscles, both compounds act antagonistically to each other. Whereas OA enhances the contraction properties of skeletal muscles, TA has the opposite effect (Saraswati et al., 2004; Selcho et al., 2012; Ormerod et al., 2013). Insect flight, which is the most energy-demanding physical activity, is also tightly controlled by OA signaling (Blau et al., 1991; Brembs et al., 2007), further showing the central role of monoaminergic neurotransmission for energy-demanding behaviors in general. Another behavior with a major impact on the balance between energy intake and expenditure is sleep, which thus takes a central position for energy homeostasis. In *Drosophila*, it was shown that the amount of sleep is directly correlated with starvation resistance (Slocumb et al., 2015). OA acts as a wake-promoting agent and impairments in the biosynthesis of OA are associated with enhanced daily sleep (Crocker and Sehgal, 2008; Crocker et al., 2010). At least in part, these effects of OA and/or TA are mediated through their modulatory action on insulin release from insulin producing neurons, which is thought to be mediated through the OAMB receptor located on these cells (Erion et al., 2012; Luo et al., 2014; Li et al., 2016). Recently, we could show that OA has a direct impact on energy expenditure-related metabolic traits, namely, it enhances the resting metabolic rate, thus reducing body fat. Consequently, reduced OA signaling leads to lowered metabolic rates and increased body fat with all its downstream consequences such as reduced life span and increased starvation resistance (Li et al., 2016). Reproduction, which critically depends on matching metabolic parameters, is also tightly controlled by OA signaling (Lee et al., 2003, 2009; Li et al., 2015), further demonstrating the role of OA to orchestrate numerous physiological actions within the organism.

Despite this body of information, we know little about the molecular mechanisms that are responsible for transducing the effects of either of these two monoamines into a suitable physiological reaction. Most importantly, the specific roles of the four OA and three TA receptors in this process remains to be elucidated (El-Kholy et al., 2015). Thus, we analyzed a set of transgenic animals impaired in expression of one of these different receptors each and employed RNAi experiments with the most relevant receptor genes targeted to major metabolic organs (brain, fat body and oenocytes).

## Materials and Methods

### Fly Stocks and Maintenance

The fly stocks used in this study were as follows: *TDC2^Ro54^* flies were generously provided by Jay Hirsh (University of Virginia, Charlottesville, VA, USA; Cole et al., 2005), *Tß*H^M18^ flies were generously provided by Henrike Scholz (University of Cologne, Köln, Germany) and OAMB-defective flies by Kyung-An Han (University of Texas, El Paso, TX, USA; Lee et al., 2003). The PromE(800)-Gal4 (oenocyte-Gal4) line was obtained from Joel Levine (University of Toronto, Toronto, ON, Canada; Billeter et al., 2009). The *octβ1R*^*f*02819^, *octβ2R*^*f*05679^, *octβ3R*^MB04794^, *TAR1*^PL00408^, *TAR2*^MB03028^ and *TAR3*^MB09692^ mutant lines used in this study were generated by the Gene Disruption Project (Bloomington Stock Center, Indiana, Bloomington, USA). The UAS-dsRNAi lines of octβ1R (#47895), octβ2R (#104050), octβ3R (#6099), TAR1 (#26876), TAR2 (#2857) were obtained from the Vienna *Drosophila* Resource Center. Other transgenic strains including nsyb-GAL4 (#51635), ppl-GAL4 (#58768), were obtained from the Bloomington *Drosophila* Stock Center. All flies, unless otherwise stated, were raised on standard yeast/cornmeal/agar medium at 25°C with about 50%–60% relative humidity under a 12 h/12 h light/dark cycle as described previously (Rahn et al., 2013; Li et al., 2015). RNAi-mediated knockdown of OAR/TARs genes in different tissues was achieved by crossing UAS-receptor RNAi line to the tissue-specific promoter GAL4 line and the F1 generation flies were kept at 29°C to enhance the RNA interference, the parental lines crossed to *w*^1118^ were used as controls.

### RT-PCR Analysis

Total RNA was extracted from the brains of 15 females kept on normal food. RT-PCR was essentially performed following recently described methods (Li et al., 2015). The following primers were used: *Rpl32* forward (5′-CCG CTT CAA GGG ACA GTA TC-3′), *Rpl32* reverse (5′-GAC AAT CTC CTT GCG CTT CT-3′), *Dilp2* forward (5′-CTG AGT ATG GTG TGC GAG GA-3′), *Dilp2* reverse (5′-ACA AAC TGC AGG GGA TTG AG-3′), *OAMB-F* (5′-CGG TTA ACG CCA GCA AGT G-3′), *OAMB-R* (5′-AAG CTG CAC GAA ATA GCT GC-3′), *Octß1R*-F (5′-GGC AAC GAG TAA CGG TTT GG-3′), *Octß1R*-R (5′-TCA TGG TAA TGG TCA CGG GC-3′), *Octß2R*-F (5′-TCC TGT GGT ACA CAC TCT CCA-3′), *Octß*2R-R (5′-CCA CCA ATT GCA GAA CAG GC-3′), *Octß*3R-F (5′-TGT GGT CAA CAA GGC CTA CG-3′), *Octß*3R-R (5′-GTG TTC GGC GCT GTT AAG GA-3′), *TAR1*-F (5′-AGA CGA GGT GCA AGG TGT TG-3′), *TAR1*-R (5′-TTC CCC GAC TTC TTT GAC TGC-3′), *TAR2*-F (5′-TGC AGT CTT TGC CAC CTT CA-3′), *TAR2*-R (5′-GTT GCC ACG AGC CTA TGA GA-3′), *TAR3*-F (5′-GAA CTT GGC CAT CAC CGA CT-3′), *TAR3*-R (5′-GTG ACG GCG AGA TAC CTG TC-3′).

### Starvation Resistance Assays

The starvation resistance assays were performed on constant conditions mentioned above. Four to five-day-old adult flies were placed in vials containing 1% agar, and dead flies were recorded every 2–3 h until all flies died. For each genotype, at least 100 flies were used in this assay.

### BODIPY Staining and Body Fat Determination

The whole fly bodies were collected and fixed in 4% paraformaldehyde for 30 min at room temperature. After washing with phosphate-buffered saline, the flies were repeatedly frozen in liquid nitrogen and thawed on ice three times, followed by staining with a solution containing 1 μg/ml BODIPY dye (Invitrogen, Darmstadt, Germany) for 1 h in the darkness before observation by epifluorescence microscopy (Olympus, Hamburg, Germany).

Total body triacylglycerols (TAGs) in flies were determined using a coupled colorimetric assay method as described previously (Hildebrandt et al., 2011; Hoffmann et al., 2013; Li et al., 2016). Briefly, eight males (or five females) per group were weighed and homogenized in 1 ml 0.05% Tween-20 using a Bead Ruptor 24 (BioLab Products, Bebensee, Germany). Homogenates were heat-inactivated for 5 min at 70°C and incubated with triglyceride solution (Fisher Scientific, Waltham, MA, USA) at 37°C for 30 min with mild shaking. The absorbance was read at 562 nm and glyceryl trioleate served as a TAG standard.

### Locomotor Activity Assay

For the negative geotaxis assay, groups of 20 flies were transferred into a 20 cm-tall glass tube without CO_2_ anesthesia and allowed to recover for 1 h. The tube was tapped three times to initiate flies to the bottom and the climbing height was photographed after 5 s. The average distance climbed in cm for each fly from five replicates was measured.

### Glucose and Trehalose Measurement

The hemolymph glucose and trehalose measurement were performed using Glucose (HK) Assay Kit (Sigma, Steinheim, Germany) with minor modifications as described previously (Li et al., 2016). The hemolymph sample was pooled from 15 flies per genotype and added to 50 μl of glucose assay reagent. After incubation for 15 min at room temperature, the glucose levels were calculated according to the standard curve established by measuring absorbance at 340 nm. For trehalose measurement, 0.25 μl of porcine kidney trehalase (Sigma, Steinheim, Germany) was added to convert trehalose to glucose. After incubation at 37°C overnight, the absorbance was measured again, and the amount of trehalose was calculated.

### Immunohistochemistry for dILP2 Measurements

Immunohistochemistry was performed as previously described (Li et al., 2016). The brains were dissected in *Drosophila* Ringer’s solution and immediately fixed in 4% paraformaldehyde in PBS for 30 min at room temperature. Subsequently, the samples were washed with PBST (0.3% Triton X-100 in PBS) and blocked in blocking-buffer (10% goat serum in PBST) for 30 min at room temperature, followed by incubation with the primary antibody (1:200 rabbit anti-dILP2, a gift from Eric Rulifson, UCSF, USA) overnight at 4°C with subsequent application of the secondary antibody (1:500 donkey anti-rabbit IgG, Jackson ImmunoLabs, Suffolk, UK) for 3 h at room temperature. After three washings, the brains were mounted on slides and images were obtained using a fluorescent microscope equipped with an apotome (Carl Zeiss Image AxioVision, Göttingen, Germany). To facilitate the quantification of dILP2 fluorescence intensities in the region of *pars intercerebralis*, series of sections were gathered under identical thickness, exposure time and all other relevant settings. Fluorescence intensity was quantified using ImageJ (National Institutes of Health, Bethesda, MD, USA).

### Statistical Analyses

All statistical analyses were accomplished using GraphPad Prism 5.0 (GraphPad Software, La Jolla, CA, USA). Starvation survivorship was analyzed by log-rank (Mantel-Cox) assays. Other parameters were evaluated using the unpaired two-tailed Student’s *t* test and one-way ANOVA. All data were presented as mean values ± SD.

## Results

**Figure 1:**
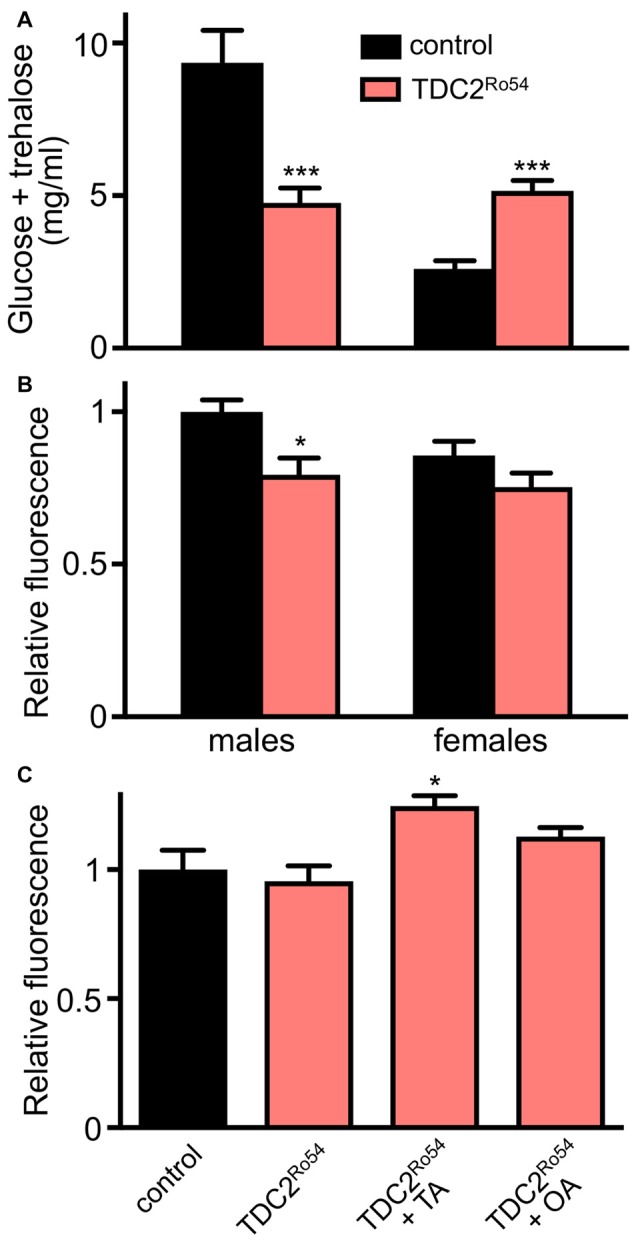
Regulation of metabolic traits in flies without octopamine (OA) and tyramine (TA; *tdc2*-deficient). Measurement of hemolymph carbohydrate levels (glucose and trehalose) in adult male and female flies of the control (w^1118^) and the TDC2^Ro54^ genotypes **(A)**. Relative fluorescence of dILP2 immunoreactive cells in the *pars intercerebralis* of control animals and TDC2^Ro54^ animals of both sexes **(B)**. Brains of female flies of control and TDC2^Ro54^ animals were analyzed as in **(B)**, but treated either with buffer, or with TA or OA prior to immunohistochemistry **(C)** (Mean values ± SD; *N* ≥ 5, *p < 0.05, ***p < 0.001).

Although TA is the biological precursor of OA, both monoamines act as independent neuroactive compounds in a wide variety of behavioral paradigms. Recently, we could show that differences between animals defective in *tβh* (TA, but no OA) and *tdc2* (no OA, no TA) could be observed regarding their body fat storage (Li et al., 2016). We analyzed these phenotypic peculiarities in more detail and could show in the current work that other metabolically relevant traits also differ between both types of animals. Whereas hemolymph carbohydrate levels are lower in both sexes of the *tβh*-defective animals, we could observe sex-specific differences in *tdc2*-defective animals where males show the same phenotype as *tβh*-defective animals did, whereas females show the opposite phenotype (Figure 1A). This sex-specific discrepancy was also observed for food intake by *tdc2*-deficient females, which show reduced food intake while males did not show these alterations. Most impressive were the differences in metabolic rates, where only OA-deficient animals show a substantial reduction, while *tdc2*-deficient ones have an unaltered metabolic resting rate (Li et al., 2016). Regarding insulin secretion, *tdc2*-deficient animals showed a slight reduction in the dILP2 content of the IPCs, which is equivalent to the situation under OA deficiency (Figure 1B). Feeding OA and TA to these animals led to a slight reduction in dILP2 release (Figure 1C).

**Figure 2:**
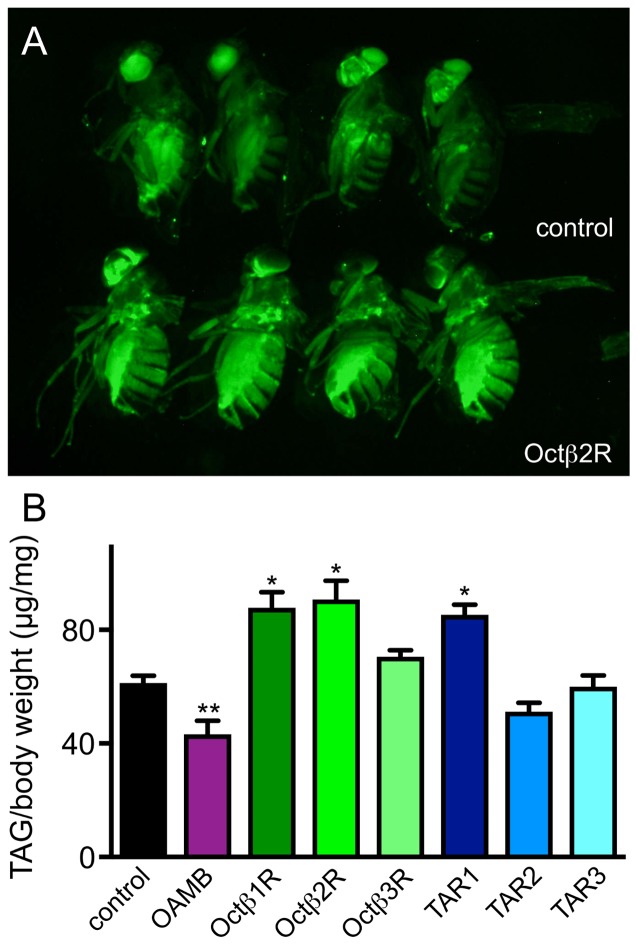
Body fat content of flies deficient in different receptors receptive for OA or TA. BODIPY staining of the *octß2r*-deficient animals (**A**, bottom) are shown in comparison with labeling of matching controls (**A**, top). Quantitative total body triacylglycerol (TAG) assay was performed with adult females of the different genotypes **(B)** (Mean values ± SD; *N* ≥ 5, *p < 0.05, **p < 0.01).

In order to learn more about their relevance for various metabolic traits, we choose a series of *Drosophila* lines carrying insertions in the respective genes coding for OA and TA receptors that should effectively impair expression of functional proteins. OA and TA transmit their effects via a total of seven G-protein coupled receptors, with four being specifically tuned to react to OA and three to TA. They can be further subgrouped regarding their primary structures into a more alpha-adrenergic subtype (OAMB), those sharing similarities with ß-adrenergic receptors (Octß1R-Octß3R) and two classes of TA receptors (Maqueira et al., 2005; El-Kholy et al., 2015). The TAR1 (also known as TyrR, Oct/TyrR) and the other two TARs (TAR2, also known as TyrR1 and TAR3) do not cluster together (El-Kholy et al., 2015). A recent analysis utilizing promoter reporter lines revealed the spatial distribution of the different receptors in the different tissues of the fly. Flies without OA (*tβh*-deficient animals) show an impressive metabolic phenotype, they are obese with fat deposits increased by more than 30% compared with matching controls (Li et al., 2016). We measured the triglyceride levels in different fly lines defective in expression of the corresponding receptors and observed that flies impaired in expression of the Octß1R, the Octß2R and the TAR1 showed a significantly enhanced fat deposition as observed by BODIPY staining of the corresponding animals, while the OAMB-deficient ones had reduced fat levels. As an example, we show the staining of the *octβ2r*-deficient flies (Figure 2A). In order to quantify this effect, we measured triglyceride levels of these animals and obtained a similar result (Figure 2B). Results from this quantitative approach were almost congruent with that obtained using the fat staining approach. As the OAMB has already been described in greater detail (Erion et al., 2012), we excluded the OAMB from all downstream studies.

**Figure 3:**
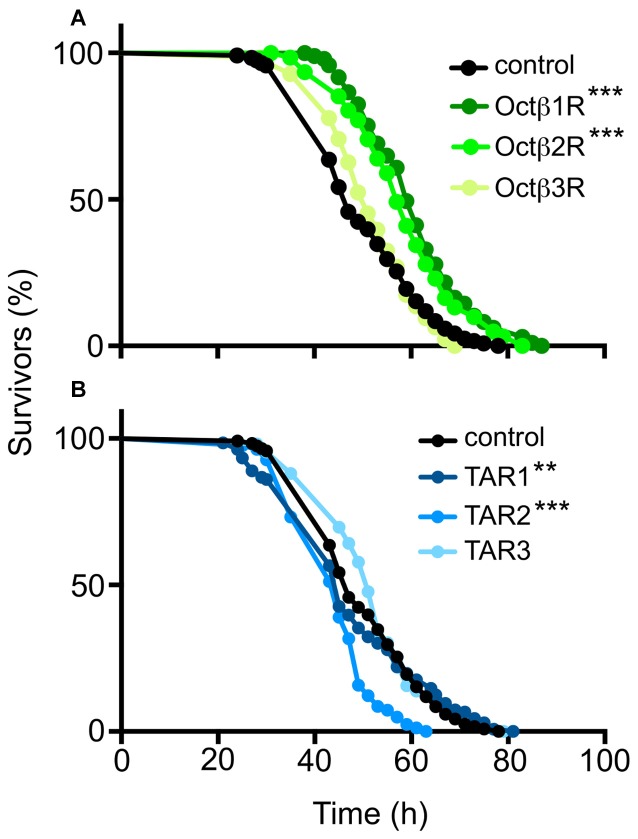
Starvation resistance of flies defective in OA or TA receptor gene expression. Those flies deficient in the expression of OA receptors **(A)** or TA receptors **(B)** were starved and the number of dead flies counted every 2 h. *w^1118^* flies served as a control. *N* = 100, **p < 0.01, ***p < 0.001.

Differential fat deposition is assumed to directly influence important metabolic traits such as starvation resistance (Ballard et al., 2008). Thus, we analyzed starvation resistance in these animals and identified significant differences to matching control populations (Figure 3). Whereas the Octß1R, Octß2R and TAR1 (Figures 3A,B) show statistically significantly increased starvation resistances, the TAR2 had a lower resistance, whereas the resistances of the Octß3R and TAR3 were not different from the controls (Figures 3A,B).

**Figure 4:**
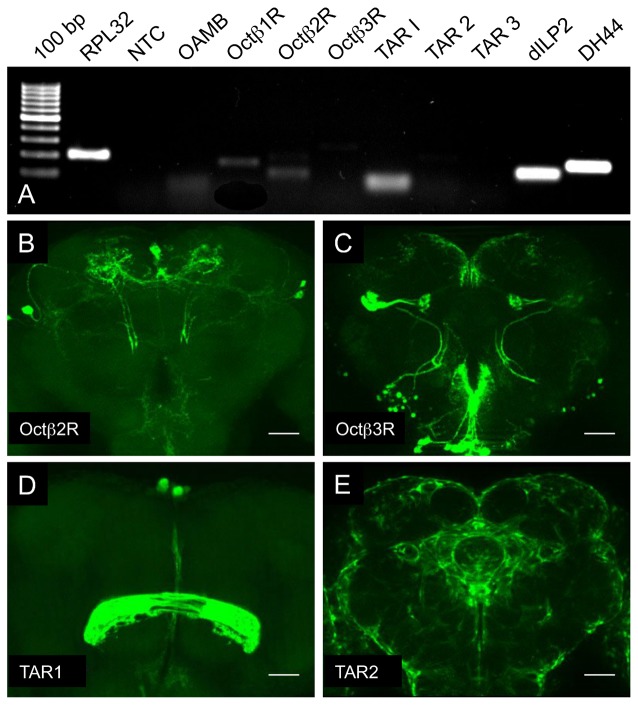
Expression analysis of OA and TA receptor genes in the *pars intercerebralis*. RT-PCR analysis of manually isolated *pars intercerebralis* areas with oligonucleotides specific for the listed genes **(A)**, NTC, no template control. Analysis of the expression patterns of fourth deficient different promoter-Gal4 lines **(B–E)**, specifically labeling cells positive for *octβ2r* **(B)**, *octβ3r* **(C)**, *tar1* **(D)**, *tar2* **(E)**. Scale bars = 50 μm.

One component that has a major effect on the fat content of flies is the level of insulin release from cells in the *pars intercerebralis*. We first analyzed which of the corresponding receptors are expressed in this peculiar brain region that contains different neurosecretory cells including those that produce and release the most important insulins in the fly (dilp2, dIlp3, dIlp5), but also those that produce, e.g., DH44, DH31 or SIFamide. Thus, we isolated the *pars intercerebralis* region manually and used the resulting material as a template for RT-PCR. From the receptors tested, the OAMB, the Octß1R, the Octß2R and the TAR1 showed specific signals, which implied that they are indeed expressed in neurosecretory cells of the brain (Figure 4A). We reanalyzed some of the doubtful candidates genes using promotor-Gal4 lines and showed expression in lateral parts of the *pars intercerebralis* for some of them, which implies that most of the OA and TA receptors are present in the *pars intercerebralis*, presumably to modulate hormone release from the corresponding cells in this highly specialized brain region (Figures 4B–E).

**Figure 5:**
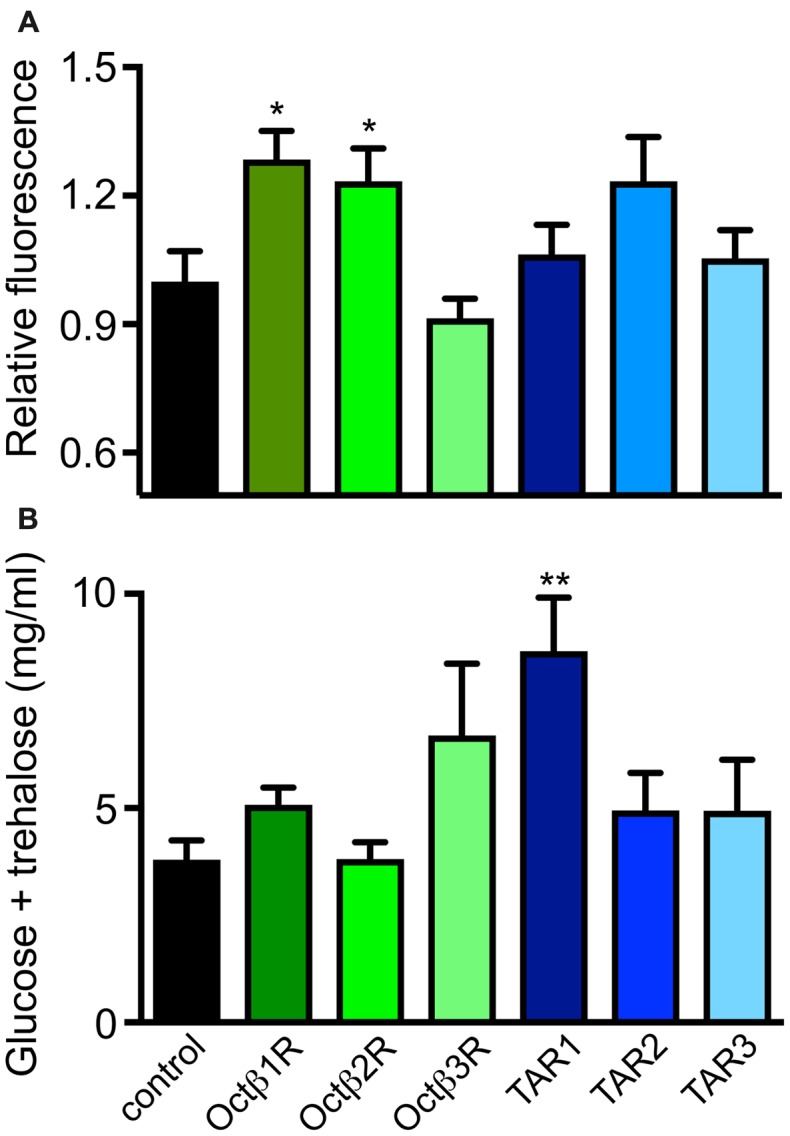
Role of different OA and TA receptors for control of insulin release. Relative dILP2 immunofluorescence was measured in *pars intercerebalis* regions of adult female flies of the indicated genotypes **(A)**. Hemolymph sugar concentrations were measured in hemolymph samples from females of the corresponding genotypes **(B)**. Mean values ± SD; *N* ≥ 5, *p < 0.05, **p < 0.01.

**Figure 6:**
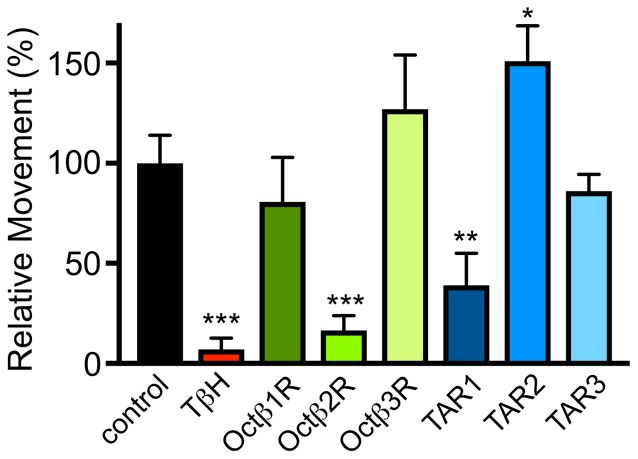
Movement ability in flies deficient in OAergic, TAergic signaling. The ability to climb a vertical plane was quantified in animals with different genotypes. The distance observed in control flies was set as 100%. Mean values ± SD; *N* ≥ 10, *p < 0.05, **p < 0.01, ***p < 0.001.

Based on this information, we analyzed the dILP2 content in insulin-producing cells of the *pars intercerebralis* of female flies of the corresponding lines (Figure 5A). Only flies with insertions in the *octß*1R or *octß*2R genes had statistically significantly different dILP2 levels in their insulin-producing cells. Directly associated with the insulin release is usually the hemolymph sugar level. We measured the combined sugar levels (glucose + trehalose) in the hemolymph of the corresponding flies and identified higher glucose levels for *octß*1R-insertion-carrying flies (Figure 5B). The control of traits that are directly associated with energy expenditure is obviously highly relevant in this context. The ability to perform complex movement tasks, such as climbing vertical planes, was addressed (Pfeiffenberger et al., 2010; Li et al., 2016). For this, we used a simple negative geotaxis assay. OA (*tβh*-defective) deficient animals showed a massively reduced ability to perform this task. While flies impaired in the *tar2* receptor showed an increased climbing activity, the *tar1*-defective lines showed reduced abilities. Most impressive were the impairments seen in lines with impaired *octß*2r expression, as they showed movement impairments that almost matched those seen in animals devoid of OA (Figure 6).

**Figure 7:**
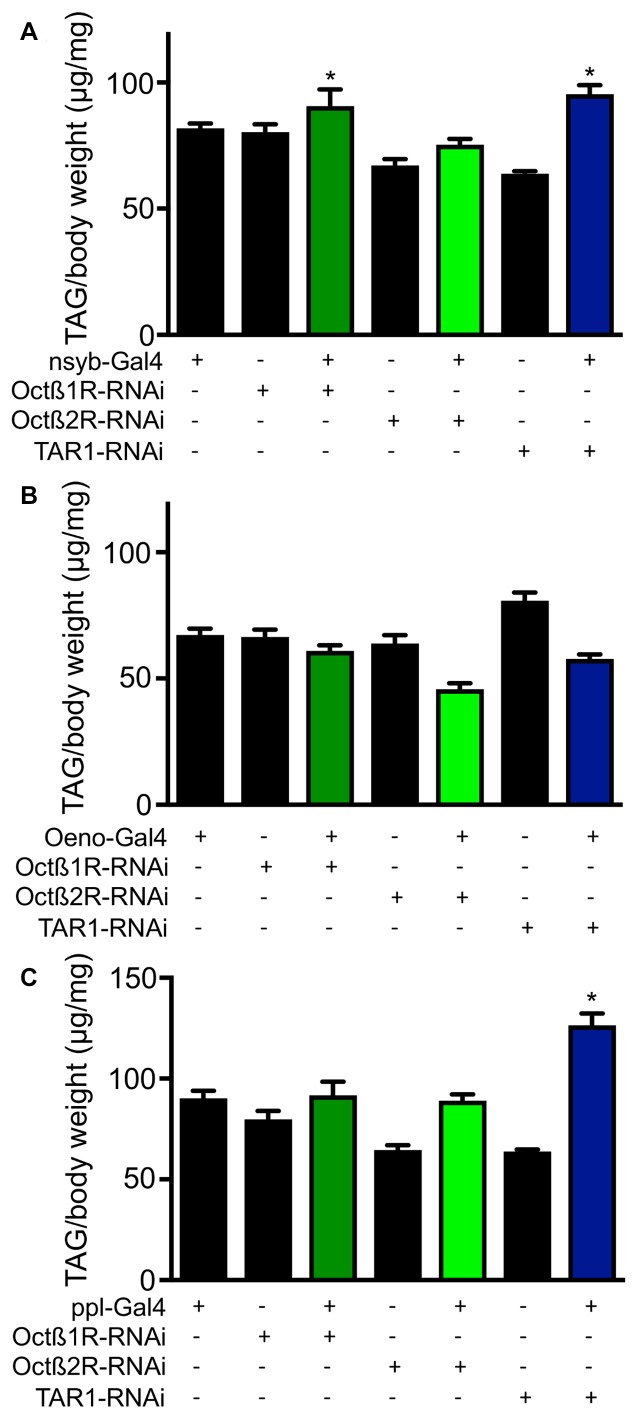
The effect of RNAi in different tissues on the fat body content. RNAi experiments as well as matching controls were performed with a neuronal driver (nsyb-Gal4, **A**), an oenocyte specific driver **(B)** and a fat body specific driver (ppl, **C**). Mean values ± SD; *N* ≥ 5, *p < 0.05.

**Figure 8:**
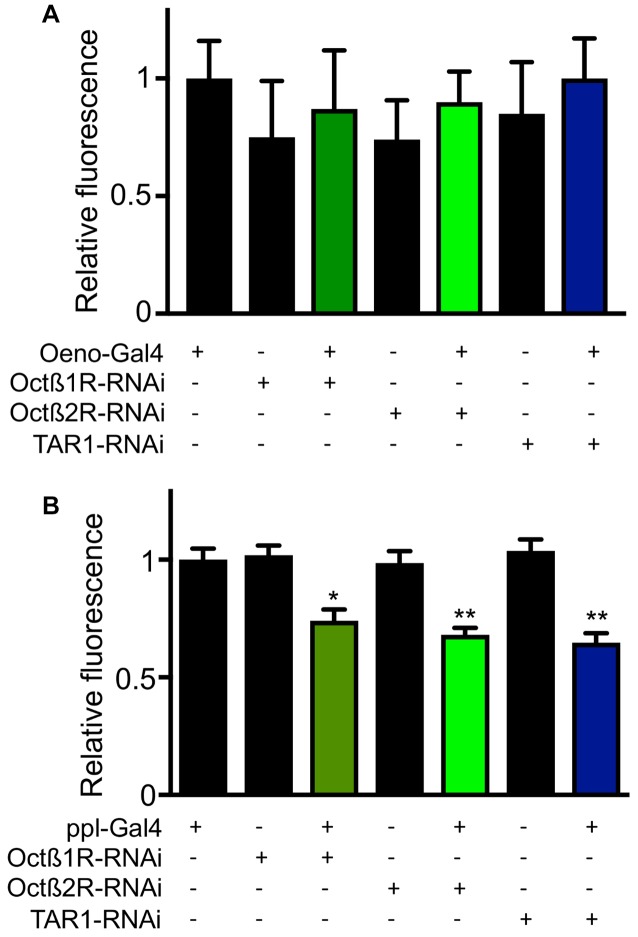
Effects of RNAi in peripheral organs for the dILP2 level in insulin producing cells. RNAi experiments as well as matching controls were performed with an oenocyte specific driver **(A)** and a fat body specific driver (ppl, **B**). Mean values ± SD; *N* ≥ 5, *p < 0.05, **p < 0.01.

In order to elucidate the mechanisms underlying differential fat contents of the different receptor-deficient lines, we performed RNAi experiments with selected receptor lines. Silencing expression in the CNS using the *nsyb* driver line revealed slightly increase body fat especially for those animals with reduced *octß*1r and *tar1* expression in the nervous system (Figure 7A). Targeting this intervention to oenocytes led to slightly reduced body fat in *octß*2r-deficient flies (Figure 7B). Silencing expression in the fat body (ppl-Gal4, Figure 7C) increased body fat only in case of *tar1* silencing (Figure 7C). Moreover, we analyzed the effects of RNAi-mediated silencing in the oenocytes (Figure 8A) and the fat body (Figure 8B) on the release of dILP2 from insulin-producing cells in the *pars intercerebralis*. If we analyzed the dILP2 concentration in insulin producing cells in response to RNAi-mediated gene-silencing of the corresponding receptor genes, we observed no changes in response to manipulation in the oenocytes (Figure 8A), but a profound reduction in response to manipulation in the fat body for *octß*1r, *octß*2r and *tar1* (Figure 8B).

## Discussion

To allow for a suitable organismal reaction in response to different internal or external situations, behavioral and metabolic traits have to be well-orchestrated. The monoamines OA and TA are central mediators of this complex interorgan communication system. Thus, they occupy the same position that epinephrine and norepinephrine take in vertebrates. Consequently, impairing their signaling properties incurs a great variety of metabolic alterations. Among these modifications, those associated with body fat stores are most conspicuous. Despite the structural similarities between OA and TA, they obviously act differentially in the regulation of major metabolic traits. Whereas animals devoid of OA (*tβh*-deficient) are obese, those without OA and TA (*tdc2*-deficient) are not, implying that the effects of both monoamines on this major metabolic trait are opposed. In order to learn more about the underlying mechanisms, we focused on the corresponding receptors, as results obtained with those animals defective in synthesis of either OA (TβH) or OA + TA (TDC2) are not easy to interpret. *Tβh*-deficient animals produce no OA, but contain higher amounts of TA, *tdc2*-defective animals, on the other hand, have neither OA nor TA, which complicates direct assignments of specific phenotypes to either OA or TA.

It was our assumption that the major metabolic phenotype of *Tβh*-deficient animals, the high fat content, is mediated via interaction with only one specific receptor, which turned out not to be the case. A total of two out of four different line defective in OA receptors (Octß1R, Octß2R) and one line defective in TA receptors (TAR1) showed an increased body fat content, thus phenocopying OA-deficient animals (see Table 1). Moreover, animals defective in expression of the *oamb* receptor gene are leaner than matching controls are. As expected, the increased body fat observed in some of the flies came with enhanced starvation resistances. This observation implies that mechanisms required to modulate body fat content are more complex than anticipated, thus mirroring the situation found in vertebrates, where regulation of body fat is mediated by sets of α- and β-adrenergic receptors rather than by only single representatives of this family.

**Table 1:** Summary of the effects seen in fly lines defective in expression of the corresponding receptors.

	OAMB	Octß1R	Octß2R	Octß3R	Tar1	Tar2	Tar3
Body fat	down	up	up	-	up	-	-
Starvation resist	n.a.	up	up	-	down	down	-
Insulin signaling	n.a.	up	up	-	-	-	-
Glucose level	n.a.	-	-	-	up	-	-
Movement	n.a.	-	down	-	down	up	-

*Significant changes in comparison to matching controls are listed as up or down. n.a. means not analyzed*.

It is not completely understood how signaling through these receptors controls body fat content. Different mechanisms have to be taken into account, comprising behaviors that are directly linked with energy intake or energy expenditure (Crocker and Sehgal, 2008; Li et al., 2016). Moreover, controlling release rates of neurohormones or conveying direct effects on peripheral organs such as the fat body or skeletal muscles may also be relevant in this context (Crocker et al., 2010; Nässel et al., 2015). Regarding the expression profiles of the different OA and TA receptors, all options listed above have to be taken into account. Among these possible actions of OA and TA, the control of insulin release is most interesting, as it would add another mechanism to the list of almost identical functions shared by OA/TA and epinephrine/norepinephrine. It has been proposed that the OAMB receptor mediates the effects of OA on insulin release via direct control of release rates (Luo et al., 2014; Nässel et al., 2015). Apparently, the situation is more complex, as the *oamb*-deficient flies are lean, although they should convey OA’s action on the IPCs. Moreover, the body fat phenotype observed in other receptor defective lines did not correlate neither with insulin release rates nor with hemolymph glucose levels. The anticipated role of OAMB as the major OA receptor operative in IPCs is still not fully supported. On the other hand, the other lines defective in expression of other OA (Octß1R and Octß2R) receptors that show enhanced body fat deposition, exhibit reduced insulin release rates, which is counterintuitive (Luo et al., 2014; Nässel et al., 2015; Li et al., 2016). The TAR1, which also is involved in body fat control has no obvious effects on insulin release, which shows that controlling insulin release by octopaminergic neurotransmission is primarily (and eventually exclusively) mediated via OA receptors.

If we take a look at the reduced ability to move in the vertical direction, the substantially reduced ability apparent in animals without OA (*TβH*-defective animals) was also observed in animals defective in expression of the *octß*2r receptor gene. Although some of the other lines defective in one of the various OA and TA receptors showed also reduced abilities to perform this behavioral task, this was in no case as severe as for the OA deficient animals. This phenotype nicely correlates with the massive expression of the *octß*2r receptor gene in skeletal muscles of larval and adult *Drosophila* (El-Kholy et al., 2015), which thus might be due to the peripheral actions of OA for controlling movement activities mediated via the Octß2R receptor.

A very complex inter-organ communication was revealed through use of tissue-specific RNAi to analyze contributions of specific OA and TA receptors for various metabolic traits. Silencing expression of specific receptor genes in neurons only (driven by *nsyb*-Gal4) revealed increased body fat only for the *oct1ß*r gene, whereas the other were almost unaffected. The lack of phenotypes observed in RNAi experiments is not easy to explain, it can result from the lack of relevance in the targeted tissue, but it can also be due to insufficient silencing that permits to uncover these relevant phenotypes.

Interestingly, silencing of some receptor genes in the fat body revealed relatively strong effects on dILP2 levels in the brain and therewith on insulin release properties. This might be an effect of remote control of insulin release by the fat body, which has already been shown to be operative in this tissue (Géminard et al., 2009; Rajan and Perrimon, 2012). The three receptors under investigation (Octß1R, Octß2R und TAR1) are all expressed in the fat body at low levels (El-Kholy et al., 2015), which making a direct interaction possible.

Taken together, we aimed to understand the various facets of OAergic and TAergic control of metabolic traits in more depth using animals with defective expression of peculiar OA or TA receptors. It became apparent that a complex network comprising different receptors in different tissues is responsible for the control of metabolic traits such as body fat content. Whereas some actions of OA and TA can be attributed to specific receptor subtypes, this is not possible for others. The reduced ability to perform complex movement tasks appears to depend on OA signaling mediated via the Octß2R in skeletal muscles. The regulation of other metabolic traits appear to be much more complex and involve complex remote control effects, which might have been expectable especially as OA as well as TA are thought to take a central role in interorgan communication.

## Author Contributions

YL, CF and TR conceived the study and wrote the manuscript, YL, LT, JF, SN, SE-K, FS performed experiments, YL, CG and IB evaluated the data.

## Conflict of Interest Statement

The authors declare that the research was conducted in the absence of any commercial or financial relationships that could be construed as a potential conflict of interest. The reviewer VM and handling Editor declared their shared affiliation, and the handling Editor states that the process nevertheless met the standards of a fair and objective review.
